# The Synthesis and Polymer-Reinforced Mechanical Properties of SiO_2_ Aerogels: A Review

**DOI:** 10.3390/molecules28145534

**Published:** 2023-07-20

**Authors:** Wang Zhan, Le Chen, Qinghong Kong, Lixia Li, Mingyi Chen, Juncheng Jiang, Weixi Li, Fan Shi, Zhiyuan Xu

**Affiliations:** 1 School of the Environment and Safety Engineering, Jiangsu University, Zhenjiang 212013, China; 1000003002@ujs.edu.cn (L.L.); 1000004815@ujs.edu.cn (M.C.); 2200904004@stmail.ujs.edu.cn (W.L.); sf_endeavor@163.com (F.S.); 2222209101@stmail.edu.cn (Z.X.); 2 Department of Electronic Engineering, School of Electronic Science and Engineering, Nanjing University, Nanjing 210023, China; chenle26@nju.edu.cn; 3 College of Safety Science and Engineering, Nanjing Tech University, Nanjing 213000, China; jcjiang_njtech@163.com

**Keywords:** silica aerogels, polymer, mechanical properties, crosslinking

## Abstract

Silica aerogels are considered as the distinguished materials of the future due to their extremely low thermal conductivity, low density, and high surface area. They are widely used in construction engineering, aeronautical domains, environmental protection, heat storage, etc. However, their fragile mechanical properties are the bottleneck restricting the engineering application of silica aerogels. This review briefly introduces the synthesis of silica aerogels, including the processes of sol–gel chemistry, aging, and drying. The effects of different silicon sources on the mechanical properties of silica aerogels are summarized. Moreover, the reaction mechanism of the three stages is also described. Then, five types of polymers that are commonly used to enhance the mechanical properties of silica aerogels are listed, and the current research progress is introduced. Finally, the outlook and prospects of the silica aerogels are proposed, and this paper further summarizes the methods of different polymers to enhance silica aerogels.

## 1. Introduction

Aerogels are the lightest solid materials in the world due to their high porosity and low density [1,2,3,4]. The liquid constituent of these materials are substituted with air and form intact interconnected solid structures. Relying on their unique mesoporous structure, aerogels demonstrate excellent properties and have been favored by researchers [5,6,7,8]. There are many kinds of aerogels, such as silica aerogels [9], carbon aerogels [10,11], polymer aerogels [12,13,14,15], metal oxide aerogels [16,17,18], metal aerogels [18], and bio-based aerogels [19,20]. These aerogels are considered as the “wonder materials” and have broad prospects of application in various fields [21,22,23,24,25].

**Figure 1:**
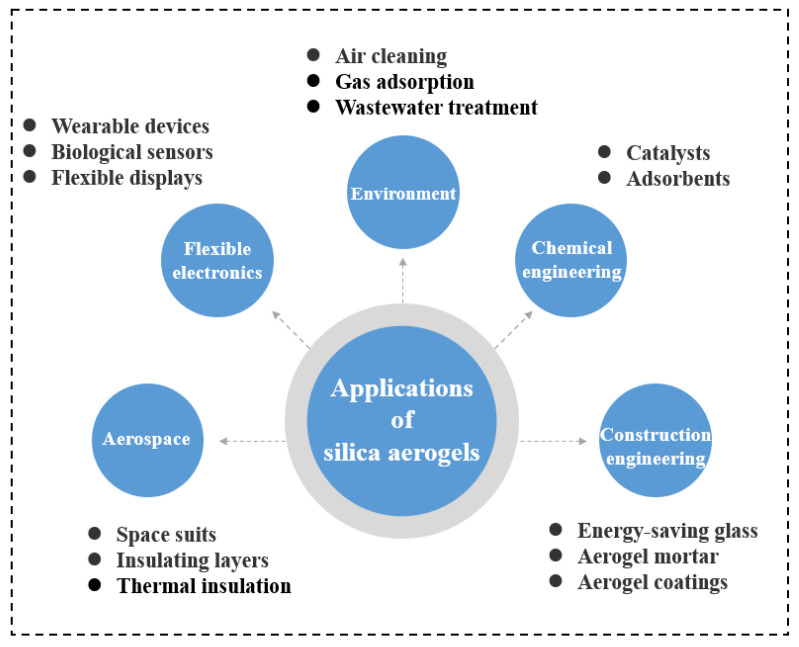
The applications of silica aerogels in various fields.

Silica aerogels with excellent properties of low bulk density (0.003~0.200 g/cm^3^), high porosity (80~99.8%), large specific surface area (500~1500 m^2^/g), and low thermal conductivity (0.015~0.030 W/m·K) are the typical representatives of aerogels [26,27,28,29]. It has been more than 90 years since silica aerogel was first invented by the American scientist Kistler in 1931 [30,31]. Although silica aerogels have a long history of development, their commercial production history only dates back to about 20 years. In 2001, Aspen realized the commercial production of silica aerogels for the first time in the United States. As Figure 1 shows, silica aerogels are widely used in construction engineering [32,33], aeronautical domains [34,35,36], environmental protection [37,38], flexible electronics [39], and chemical engineering [40,41,42]. Especially in the aerospace field, silica aerogels have made remarkable contributions to the safety of personnel and equipment [43].

However, the application of silica aerogels on a large scale is still limited due to the poor mechanical properties of silica aerogels [44]. The main reason for the poor mechanical properties of silica aerogels is their pearl-necklace-like three-dimensional network structure. This network structure is connected by the interparticle necks, and the connection strength is very fragile. However, other properties of silica aerogels are excellent in the engineering field. Thus, the problem of the poor mechanical properties of silica aerogels urgently needs to be solved [45,46]. 

**Figure 2:**
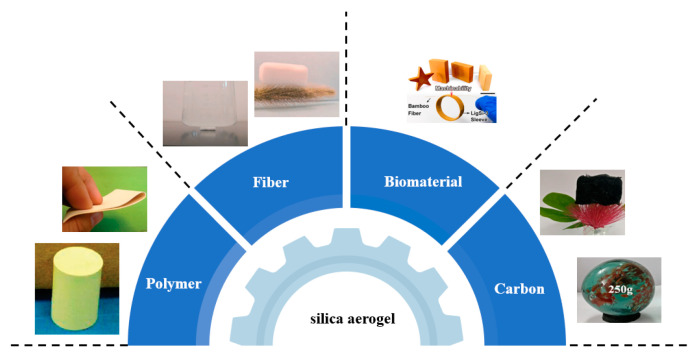
Materials for enhancing mechanical properties of silica aerogel. Reprinted with permission from Refs. [49,50,51,52]: Copyright 2019, Elsevier; Copyright 2022, Elsevier; Copyright 2011, American Chemical Society; Copyright 2022, American Chemical Society.

The secondary particles of silica aerogels are connected via point contact with a small contact area and weak bond force between the particles [47]. Once the aerogels have suffered from external stress, the neck region between the secondary particles break, resulting in the connection being disconnected and the gel skeleton collapsing [48]. In order to solve the above problems, scholars around the world have conducted extensive research. Focusing on the problem of the poor mechanical properties of silica aerogels, reinforcing phases have been introduced to improve the mechanical properties of silica aerogels. As shown in Figure 2, the reinforcing phases mainly include carbon, biomaterial, fiber, and polymer. The mechanical properties of silica aerogels have been enhanced to varying degrees through the experiment.

In this paper, the composites of silica aerogels with polymers are summarized, and the properties are discussed in detail.

## 2. Synthesis of Silica Aerogel

**Figure 3:**
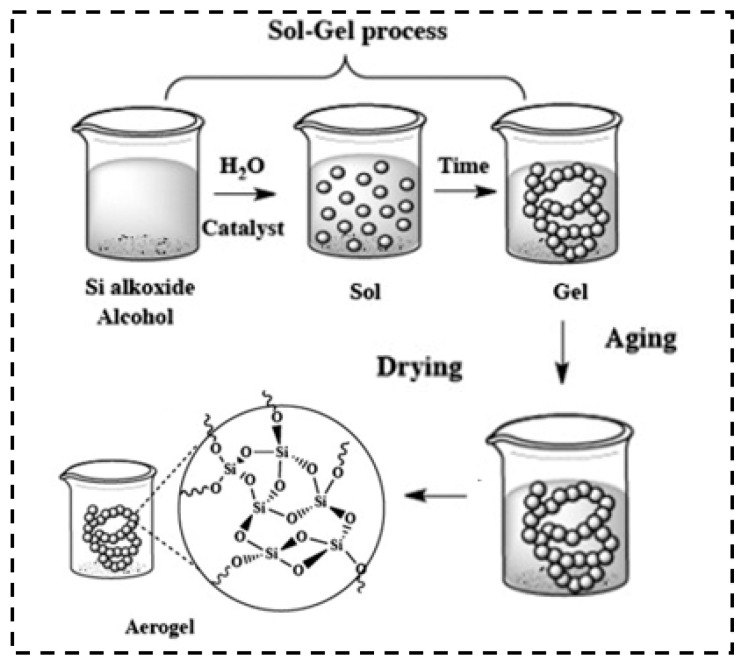
The preparation of aerogel using sol–gel process. Reprinted with permission from Ref. [54]: Copyright 2014, Elsevier.

As shown in Figure 3, the preparation of the silica aerogels comprises (a) synthesis, (b) aging, and (c) drying [53]. The synthesis of silica aerogels mainly depends on the method of sol–gel [54]. During the sol–gel process, a three-dimensional network structure is built [55,56]. And the properties of silica aerogels are influenced by the precursors, catalysts, temperature, surface treatments, mass concentration, pH, and drying [57,58,59,60]. Then, the aging process enhances the network structure of the silica sol. Finally, the solvents are removed from the solid via the drying technology. 

### 2.1. Sol–Gel Chemistry

**Figure 4:**
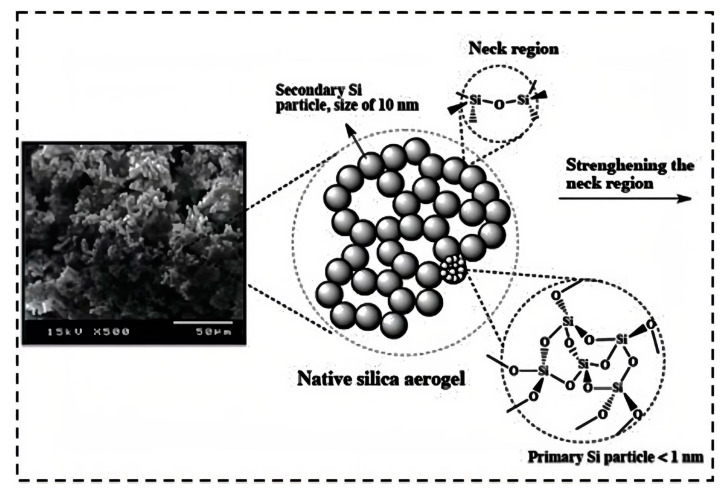
Primary and secondary silica particles of the silica aerogel. Reprinted with permission from Ref. [54]: Copyright 2014, Elsevier.

Gels are colloids that are composed of colloidal particles suspended in a solvent. The sol–gel method is a common technique used for the synthesis of wet gels, and then the silica aerogels are produced via the drying process [61,62]. During the sol–gel process, ethanol or methanol is used as a solvent. Using the solvent, the precursors form a soluble gel through hydrolysis and polycondensation reactions [63,64]. The results of the reactions are greatly influenced by the pH of the solution, the concentration of silicon precursor, the reaction time, and other factors. Moreover, the solid network of silica is formed in this process, and the particles are more closely connected [65,66,67,68]. However, the repetitive washing and the tedious water-to-alcohol solvent exchange still need more time [69]. As shown in Figure 4, the primary particles form secondary particles in the solution and then the secondary particles form a continuous solid network connected by the neck regions [70]. With the development of sol–gel chemistry, silica alkoxides are the precursors of silica aerogels, such as tetraethylorthosilicate (TEOS), tetramethylorthosilicate (TMOS), and so on [71,72,73]. These precursors have a great influence on the morphology and properties of silica aerogels [74]. Moreover, various precursors can also be used together to realize the self-reinforcement of silica aerogel skeletons [75,76]. Bhagat et al. [77] applied nine different co-precursors to prepare TEOS-based silica aerogels and investigated their physical properties. The pre-polymerization of silicon precursors is also a method of silica self-reinforcement that is currently being explored. Zu et al. [78] reported a new method of pre-polymerized silica-based precursors and enhanced the flexibility of aerogels. Table 1 lists some common precursors that are used for the synthesis of silica aerogels. 

**Table 1:** Common silica precursors for silica aerogel synthesis.

Silica Precursor	Chemical Formula	Abbreviation	Physical Properties	Mechanical Properties	Thermal Properties	Ref.
Tetraethylorthosilicate	Si (OC_2_H_5_)_4_	TEOS	/	G modulus: 10.7 MPa	/	[79]
Tetramethylorthosilicate	Si (OCH_3_)_4_	TMOS	Skeletal densities: 2.2 g/cm^3^	/	/	[80]
Trimethylchlorosilane	Si (CH_3_)_3_Cl	TMCS	Surface area: 914.4 m^2^/g; porosity: 96.16%	/	/	[81]
Methyltrimethoxysilane	Si (OCH_3_)_3_CH_3_	MTMS	Shrinkage: 3.5%	/	/	[82]
Methyltriethoxysilane	Si (OC_2_H_5_)_3_CH_3_	MTES	Density: 0.1 g/cm^3^; porosity: 95.5%	Unrecoverable strain loss: 10%	Thermal conductivity: 0.038 W/m·K	[83]
Aminopropyltrimethoxysilane	H_2_N (CH_2_)_3_Si(OCH_3_)_3_	APTMS	/	/	Young’s modulus: 14 MPa	[84]
Aminopropyltriethoxysilane	H_2_N (CH_2_)_3_Si(OC_2_H_5_)_3_	APTES	Surface area: 150.9 m^2^/g	Young’s modulus: 18 MPa	Thermal conductivity: 0.037 W/m·K	[85]
Propyltriethoxysilane	C_9_H_22_O_3_Si	PTES	Density: 0.172 g/cm^3^; porosity: >90%	Elastic module: 0.35 MPa	/	[86]
Vinyltrimethoxysilane	H_2_C=CHSi(OCH_3_)_3_	VTMS	/	Elongation at break: 40~50%	Thermal conductivity: 0.06 W/m·K	[87]
Vinyltriethoxysilane	C_8_H_18_O_3_Si	VTES	Surface area: 321 m^2^/g	Compressive stress: 0.571 MPa	Thermal conductivity: 0.024 W/m·K	[88]
3-glycidoxypropyltrimethoxysilane	C_9_H_20_O_5_Si	GPTMS	/	/	Thermal conductivity: 0.032 W/m·K	[89]
Bis [3-(triethoxysilyl)propyl]disulfide	C_18_H_42_O_6_S_2_Si_2_	BTSPD	Density: 0.21 g/cm^3^; porosity: 85.5%	Young’s modulus: 2.1 MPa	/	[90]
1,6-bis(trimethoxysilyl)hexane	C_12_H_30_O_6_Si_2_	BTMSH	/	Strain: 50%	/	[91]
Bis(trimethoxysilylpropyl)amine	C_12_H_31_NO_6_Si_2_	BTMSPA	Density: 0.308 g/cm^3^; porosity: 78%; surface area: 325 m^2^/g	Shrink: 11%, compression Modulus: 15 MPa	/	[92]
Dimethyldiethoxysilane	C_6_H_16_O_2_Si	DMDES	Density: 0.082 g/cm^3^; surface area: 162.1 m^2^/g; porosity: 94.2%	/	Maximum degradation rate: 150 °C	[93]

### 2.2. Aging

Aging is an important step in strengthening the mechanical properties of silica aerogels [94]. The aging reaction principle is ascribed to accelerating the movement of sol particles and increases the probability of collision, adding the number of siloxane connections. In the aging process, the number of siloxane linkages between particles can be increased using two different mechanisms simultaneously; thus, the mechanical properties of silica aerogels will be strengthened [95]. These two different mechanisms mainly include dissolution and re-precipitation [96]. Moreover, new monomers are transported to the neck region between the particles and form the network [97]. As an important chemical bond connecting the particles together, the O-Si bonds have a great relationship with the time and temperature of the reaction. The number of the bonds affects the mechanical properties of the silica aerogels. He et al. [98] reported that a controlled temperature and pressure can enhance the mechanical properties during the process of dissolution and reprecipitation.

However, this process requires a lot of time, and the parameters of the aging process are difficult to control. During the aging process, the silica aerogels occur different degrees of shrinkage, leading to an increase in density.

### 2.3. Drying

Drying is the procedure that transports the wet silica gel to silica aerogel [99]. In this process, gas replaces the liquid in silica wet gel, and ultimately, the solid consisting of the silica network forms the aerogel. Due to differential shrinkage, warping and cracking often occur. However, previous studies have shown that the phenomena can be prevented by controlling the drying process. At present, there are three common drying methods as follows: ambient pressure drying [100,101], freeze drying [102,103], and supercritical drying [104].

Compared with the other two drying methods, the ambient pressure drying method is widely used mainly due to its lower energy consumption and because it does not require high pressure conditions [105,106,107]. However, there are still drawbacks to ambient pressure drying. The most significant disadvantage is that ambient pressure drying may be affected by capillary force, and thus, still result in collapsing or cracking [108]. Freeze drying is the technology that freezes the liquid in the wet gel into a solid and then converts the solid into a gas. In order to reduce the possibility of breakage caused by ambient pressure drying, freeze drying is used to manufacture various types of aerogels. Compared with the above methods, supercritical drying is considered as the most appropriate method to prepare aerogels. It minimizes the aerogel cracking caused by shrinkage. The liquid in the pores is transformed into supercritical fluid via supercritical drying. In this state, the surface tension of the liquid disappears completely and there is no capillary force. Finally, the supercritical fluid can be separated from the solid at a temperature above the critical temperature of the liquid [109]. At present, the more mature supercritical drying technology is low temperature drying using carbon dioxide. However, this method has high requirements for synthesis equipment and the operating environment [110].

## 3. Polymer-Modified Silica Aerogel Composites

Based on existing reports, the polymer matrix is composed of thermoplastics and thermosetting resins [111]. Polymers demonstrate excellent mechanical properties, generally with high elastic deformation and viscoelasticity [112]. In addition to preparing polymer aerogels, polymers can also be combined with silica aerogels to prepare hybrid aerogels [113]. The process of polymerization increases the chemical bonds of O–Si to enhance their mechanical properties [114].

In the past two decades, researchers believed that combining polymer and silica is an effective way to enhance the mechanical properties of aerogels [115,116,117,118]. Meanwhile, the results indicate that the interfaces between silica gel particles and polymers also have a great influence on aerogel properties [119]. In 1994, Novak et al. [120] prepared hybrid aerogels via the method of pre-synthesized polymers or in situ polymerizing species, achieving an enhanced flexibility and compressive strength through the adsorption of energy with silica aerogels compounding the polymers [121,122]. As reported in the previous research, polymer-crosslinked aerogels were studied via the following three technical approaches: (a) modifying the surface of nanoparticles to enhance the aerogel skeleton; (b) applying different types of crosslinking agents; (c) creating the network morphology of the aerogels [123,124].

**Figure 5:**
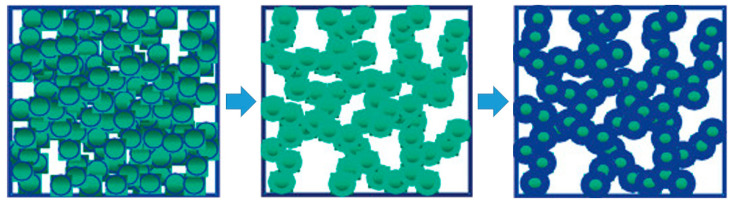
Technical approaches to improve the mechanical properties of aerogels. Reprinted with permission from Ref. [125]: Copyright 2011, American Chemical Society.

Using the method of chemical crosslinking, the polymer conformally coats the skeletal framework of the aerogels and maintains the original shape of the mesopores to reinforce their mechanical properties. As shown in Figure 5, with the addition of the polymer, the density of the aerogel will show an increasing trend. Through the growth mechanism, the number of connecting points between the secondary particles will increase, and finally resulting in the polymer-reinforcement.

Although the mechanical properties of aerogels have been improved, their density and thermal conductivity have been increased as well, and the addition of the polymers even reduce their resistance to high temperatures [126]. Using well-controlled polymerization techniques, atom transfer radical polymerization can effectively enhance the performance of aerogels without significantly increasing their density. Moreover, the aggregation and poor interfacial interaction can be solved via the combination of silica aerogels and polymer [127]. The reported polymer-reinforced aerogels are listed in Table 2, and the contents are introduced in further detail.

### 3.1. Epoxide

The mechanical properties of silica aerogels can be enhanced via epoxide. The functional groups of the epoxide can react with the amino groups on the surface of the gel skeleton. Thus, the addition of epoxide in the matrix of the silica aerogel can change its fragile properties and enhance its mechanical properties [151]. Thus, researchers have focused on applying epoxide to improve the mechanical properties of silica aerogels.

**Figure 6:**
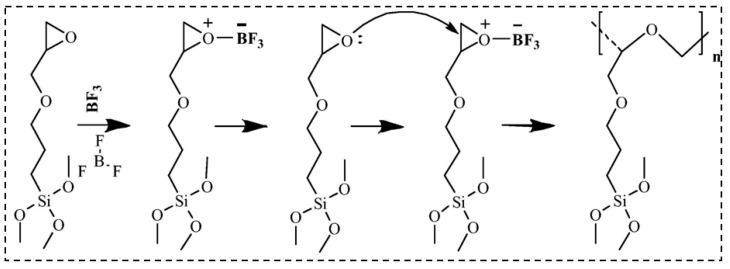
Polymerization reaction of GPTMS and molecular structure of PGPTMS. Reprinted with permission from Ref. [128]: Copyright 2020, Elsevier.

Rezaei et al. [128] have shown a new method to prepare the hybrid silica aerogel with the insulative and flexible properties. As shown in Figure 6, the researchers applied an epoxide ring containing the silica precursor and inserted flexible ether groups into the main chain using the method of ring-opening polymerization. The brittleness properties of the silica aerogels were enhanced due to the non-particulate structure. The results demonstrate that the elastic deformation of the aerogel was increased to 15%, and the mechanical properties were proportional to the density. Moreover, the aerogels have superinsulation properties with a thermal conductivity of only 0.0159 W/m K.

**Figure 7:**
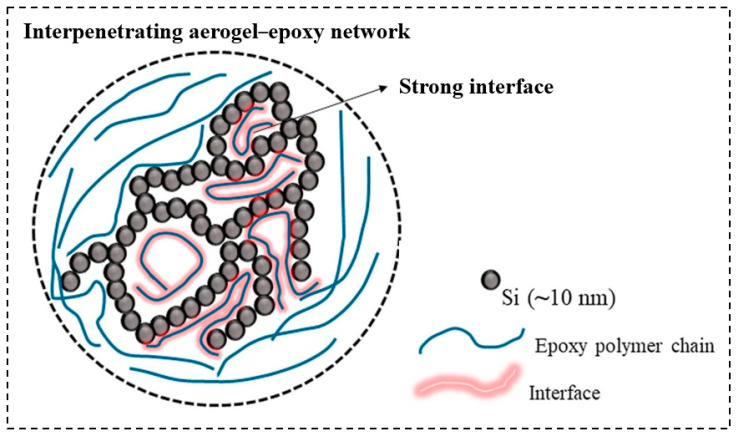
The structural model of the “interpenetrating organic-inorganic network”. Reprinted with permission from Ref. [129]: Copyright 2018, Elsevier.

Salimian et al. [129] prepared the silica aerogel–epoxy nanocomposites and investigated the fracture and toughening mechanisms. By analyzing the mechanical and thermal properties, the results suggest that the viscosity of the nanocomposite suspension was increased from 1% to 6% with the silica aerogel addition. In addition, the storage modulus, *Tg*, Young’s modulus, tensile strength, and toughness were increased by 11%, 5 °C, 35%, 62%, and 126%, respectively. As Figure 7 shows, the epoxy polymers are infiltrated into the mesopores of the silica aerogel. The fracture and toughening mechanisms are explained by the (a) crack pinning and deflection and the (b) plastic deformation.

**Figure 8:**
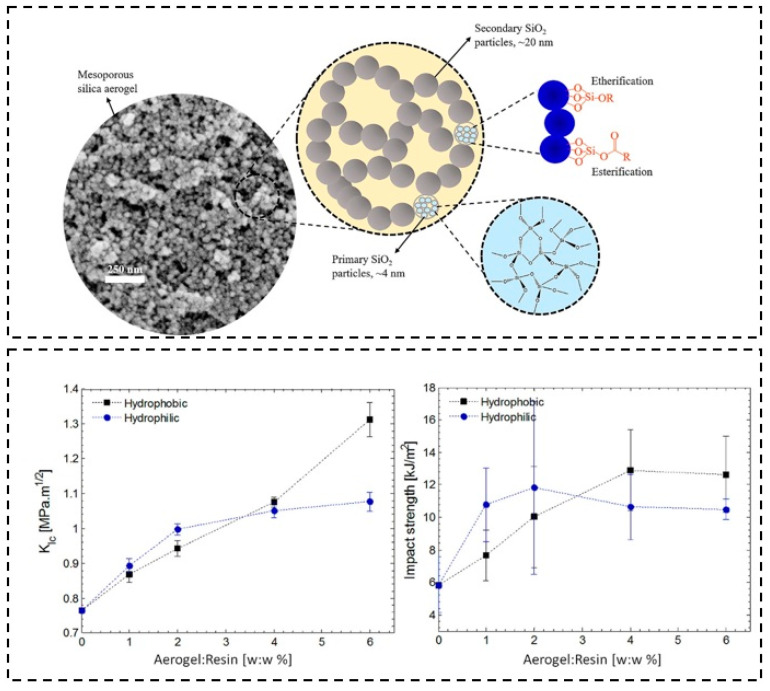
Schematic illustration of the silica aerogel particle network structure and possible covalent interactions between the epoxy and silica surface. Reprinted with permission from Ref. [130]: Copyright 2018, American Chemical Society.

Salimian et al. [130] prepared the epoxy nanocomposites using two different types (hydrophobic and hydrophilic) of silica aerogels. As shown in Figure 8, the ≡Si−O−C≡ bonds are formed between the silica surface and the epoxy polymer network. Using this method, the storage modulus, viscosity, *Tg*, fracture toughness, and impact strength were enhanced. Moreover, the fracture toughness (*K*_lc_) and impact strength increased with the increase in the hydrophobic aerogel content.

Albooyeh et al. [131] studied the influences of silica aerogel on the mechanical, vibrational, and morphological properties of epoxy. The tensile, bending, compressive, dynamic mechanical thermal analyses, and a series of tests were conducted to verify the Euler–Bernoulli beam theory. The results indicate that silica aerogels can effectively reduce the density of materials. Meanwhile, the tensile, flexural, compressive modulus and hardness of the materials significantly increased when the addition of silica aerogel was 4%.

According to the experimental data, the researchers found that the flexibility and robustness of the pore structure can be enhanced via the combination with polymers. Domènech et al. [132] synthesized a porous organic–inorganic hybrid material composed of silica and epoxy resin via a one-pot sol–gel process and subsequent supercritical drying. These results prove that controlling the bridged alkoxide proportions to enhance the mechanical properties of the silica aerogel is feasible and that the strain at 18 N can achieve 80%.

Selay et al. [133] applied silica aerogel powders with ionic liquid as a nanofiller to prepare the nanocomposite. The silica aerogels with ionic liquid possessed a lower density (0.16 g/cm^3^), higher porosity (93%), and higher thermal stability (400 °C). Moreover, the composites (silica aerogel with 1wt% ionic liquid) demonstrated better mechanical properties, such as modulus of elasticity (4156.27 MPa) and tensile strength (51.96 MPa).

Epoxide is also a common gelation initiator. He et al. [134] applied epoxides as gelation accelerators to prepare the ZrO_2_–SiO_2_ aerogels via aging and supercritical drying. The results demonstrate that the epoxides can accelerate the gelation of sol. Considering that the decomposition of the polymer leads to a decrease in the high temperature resistance of aerogels, mullite fibers were introduced as the skeletons for the aerogels. The compressive strength of the M/ZrO_2_–SiO_2_ aerogel reached 0.438 MPa and the thermal conductivity was only 0.027 W/m·K. Selver et al. [135] investigated the influences of epoxy on the mechanical, nondestructive, and thermal properties of silica aerogel composites. The results show that the epoxy composites with a 1% addition of silica aerogel exhibited better flexural strength, impact, and energy absorption. However, the thermal conductivity of the 1% silica aerogel composites increased due to the void inside of the epoxy resin being filled up.

### 3.2. Polyurea

Polyurea consists of aromatic isocyanate segments and soft polyamine chains, which are synthesized via the reaction of the amino compound with the isocyanate component. This material has excellent anti-corrosion, waterproof, and mechanical properties. Polyurea-crosslinked silica-based aerogels have the characteristics of being nano-porous and mesoporous, so they can exhibit unique thermal management behavior.

**Figure 9:**
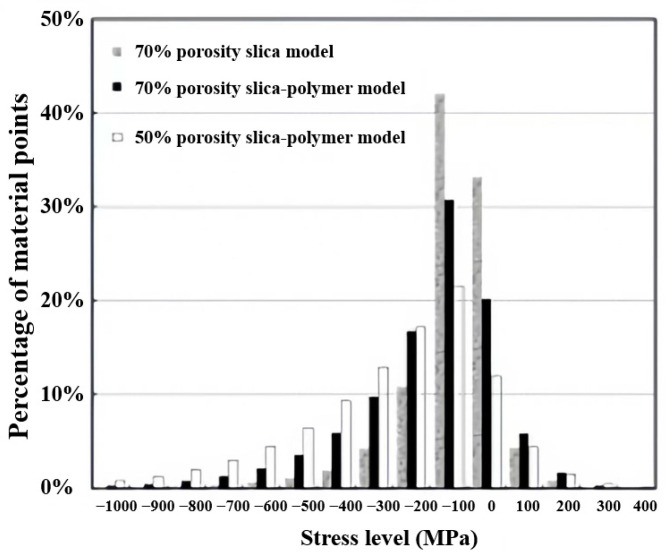
Histograms of the distribution of material points with different stress levels at 19% compressive strain deformation. Reprinted with permission from Ref. [152]: Copyright 2011, Elsevier.

Fu et al. [152] used the material point method (MPM) to study the mechanical behavior of the silica aerogels whose skeletal framework was coated by the polyurea at high strain rates. The researchers found that the MPM can model the compression of complex mesoporous structures and that the conformal polymer coating has a reinforcing function. The histograms of the distribution of material points versus the stress level at 19% strains is shown in Figure 9; the data show that the model with a 50% porosity has a wider range and exhibits more material points when under stress.

Churu et al. [136] also investigated the mechanical properties of the polymer-crosslinked templated silica aerogel (CTSA). The results show that the 1,3,5-trimethylbenzene (TMB) and triblock copolymer both have influences on the morphology of the aerogels, resulting in a change in the mechanical properties. The researchers further revealed the intrinsic relationship between the morphology and mechanical properties.

**Figure 10:**
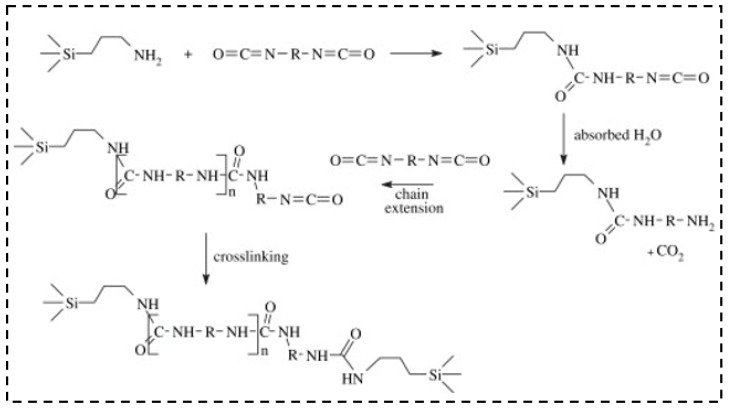
Synthetic route for amine-modified aerogel crosslinked with diisocyanates. Reprinted with permission from Ref. [137]: Copyright 2006, Elsevier.

Capadona et al. [137] reinforced the silica skeleton via the polymerization of the di-isocyanate using the amine-modified surface of a sol–gel-derived mesoporous silica network. Through the reaction with amines and urea linkages, the polymer was coated to the surface of the aerogel skeleton, which is shown in Figure 10. The results reveal that the highest density crosslinked aerogel had the highest stress at failure, exhibiting the highest modulus and crosslink.

**Figure 11:**
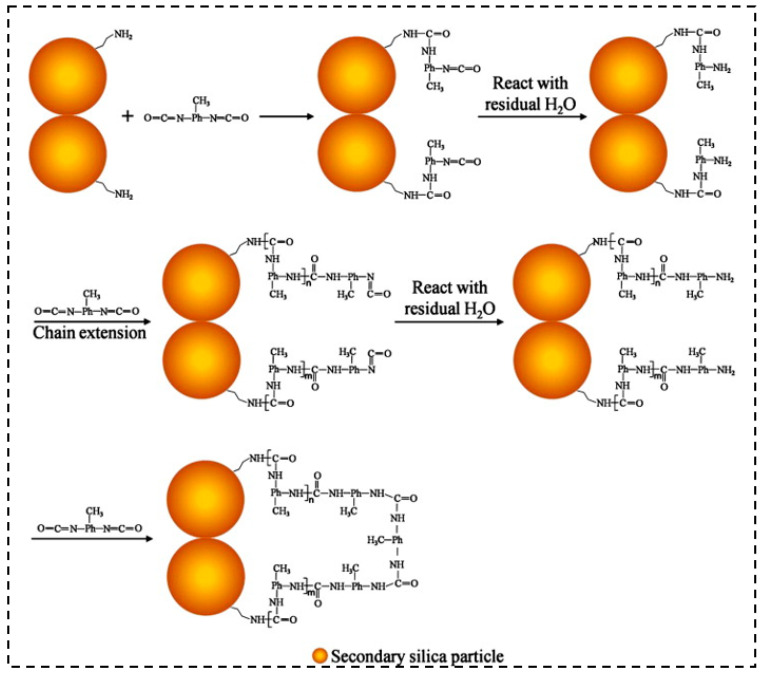
Reaction–modification of amine-modified aerogel using toluene diisocyanate. Reprinted with permission from Ref. [138]: Copyright 2011, Elsevier.

As Figure 11 shows, Yang et al. [138] prepared the modified silica gels using the method of copolymerizing tetraethylorthosilicate with 3-aminopropyltriethoxy-silane. During the ambient pressure, the researchers successfully controlled the shrinkage of the silica aerogels. The experimental data demonstrate that the elastic modulus of the silica aerogel skeleton increases because of the incorporated polymers.

### 3.3. Polyurethane

Polyurethane (PU) is a typical foaming material, and its high thermal conductivity is an important issue that restricts sustainable development [153]. Based on the previous reports, the polyurethane-based hybrids prepared using the sol–gel approach showed excellent thermal insulating effectiveness and mechanical properties due to the inorganic and organic co-networks [154]. In general, there are a certain amount of hydroxyl groups remaining on the surface of the solid skeleton of the silica wet gel. Therefore, polyurea can form covalent bonds with silica wet gel and enhance the adhesion on the surface of the solid skeleton.

**Figure 12:**
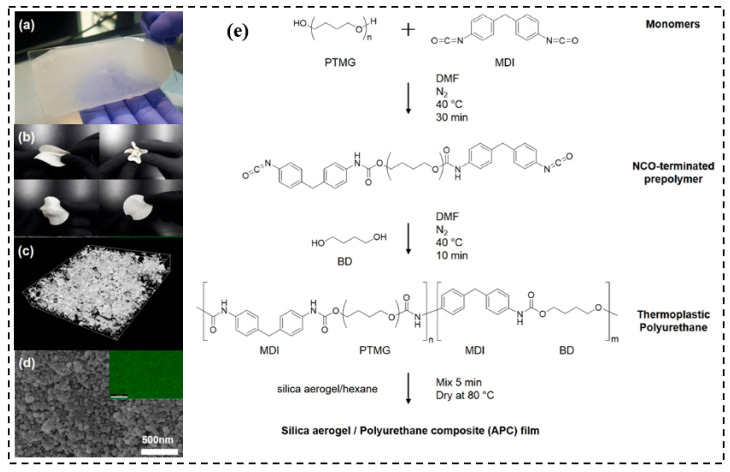
The photographs of (**a**) APC; (**b**) mechanical properties test; (**c**) m-CT image; (**d**) SEM and EDS image; (**e**) schematic of PU synthesis and fabrication process of APC. Reprinted with permission from Ref. [140]: Copyright 2019, Elsevier.

Cho et al. [140] prepared fabricated foldable silica aerogel/polyurethane composites (APCs), and the properties of the composites were theoretically verified via the proposed model. Figure 12 summarizes the morphological analysis of the PU1000 series, the schematic of the PU synthesis, and the fabrication process of the APCs. In Figure 12, the isocyanate-terminated prepolymers were synthesized by reacting with poly(tetramethylene ether glycol) (PTMG) and 2,4-diphenylmethane diisocyanate (MDI). Then, the prepared isocyanate-terminated PTMGs were chain-extended using 1–4 butanediol (BD). The results show that with an aerogel addition of 30%, the thermal conductivity of the APCs decreased by 72%, and the PU with a longer soft segment length demonstrated no breakage after bending.

**Figure 13:**
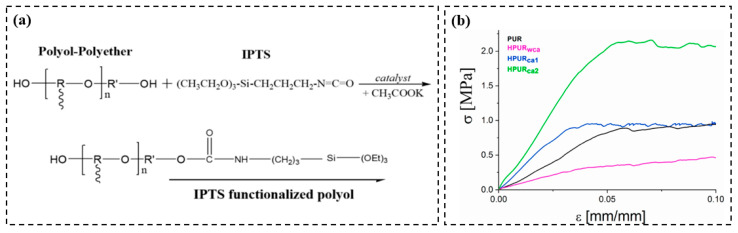
(**a**) Functionalization reaction between polyol-polyether and IPTS; (**b**) stress–strain curves of the foams. Reprinted with permission from Ref. [139]: Copyright 2021, Elsevier.

Verdolotti et al. [139] synthesized organic–inorganic polyurethane-based hybrids, leading to an enhancement of the mechanical properties and thermal insulation via the isocyanate functional groups of IPTS reacted with OH of polyol under urethane bonds, as shown in Figure 13a. The researchers investigated the influences of mechanical behaviors on the aerogel-like siloxane domains. Figure 13b demonstrates the stress–strain curves of the foams (HPUR_ca1_ and HPUR_ca2_) compared to the pristine PUR. The Young’s modulus of the foams (HPUR_ca1_ and HPUR_ca2_) achieved 30.17 MPa and 50 MPa. Moreover, the yield strength of these materials achieved 0.093 MPa and 2.15 MPa, respectively.

**Figure 14:**
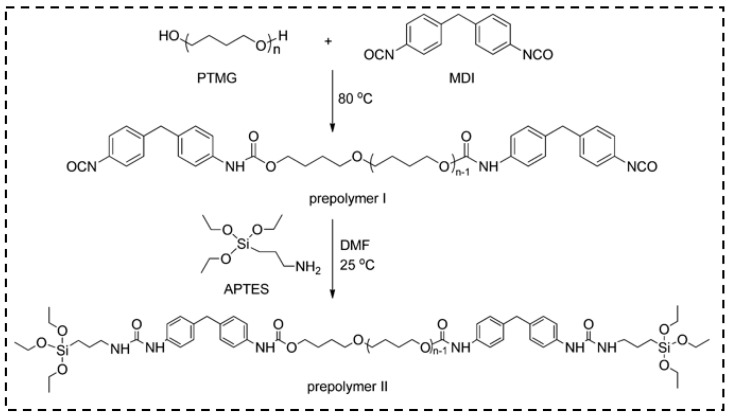
Synthesis of urethane prepolymer (prepolymer I) and APTES-end-capped prepolymer (prepolymer II). Reprinted with permission from Ref. [141]: Copyright 2013, American Chemical Society.

**Figure 15:**
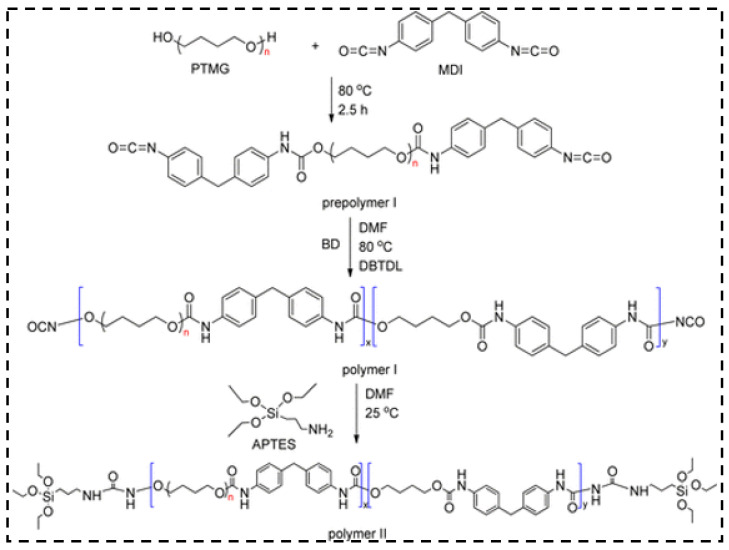
Synthesis of MDI-end-capped polyurethane (polymer I) and APTES-end-capped chain-extended polyurethane (polymer II). Reprinted with permission from Ref. [141]: Copyright 2013, American Chemical Society.

The researchers found that the flexibility of the hybrid aerogels are enhanced by the long-chain polymer molecules. Based on this theory, Duan et al. [141] prepared the mechanically reinforced hybrid silica aerogels using silane-end-capped urethane prepolymer and chain-extended polyurethane. The synthesis of prepolymer I, II, and polymer I, II are shown in Figure 14 and Figure 15, respectively. The figures mainly explain the silane end groups participating in the silica network formation and the method that controls the amounts of polyurethane added to the aerogel network. The results show that the mechanical properties were enhanced via chain-extended polyurethane and the aerogels can suffer a 70% compressive strain with the addition of polymers.

The PU foams not only have lightness properties, but also demonstrate a continuous solid network, which can be used as reinforcements. Merillas et al. [142] reinforced the silica aerogel composites using reticulated polyurethane (PU) foams via ambient pressure drying and supercritical drying. Hexamethyldisilazane (HMDZ) was used to modify the surface of silica aerogels and the continuous network hybrid aerogel was formed using polyurethane. The results show that the elastic modulus increased from 130 to 450 kPa and the thermal conductivity was as low as 0.014 W/m·K.

### 3.4. Polyimide

Compared with other organic constituents, the imide ring demonstrates a higher initial decomposition temperature [155]. Moreover, the chain structure constructed by the imide rings also create a polyimide (PI) with superior mechanical properties and a favorable chemical resistance [156]. Therefore, it is feasible to use polyimide to enhance the mechanical properties of silica aerogels.

**Figure 16:**
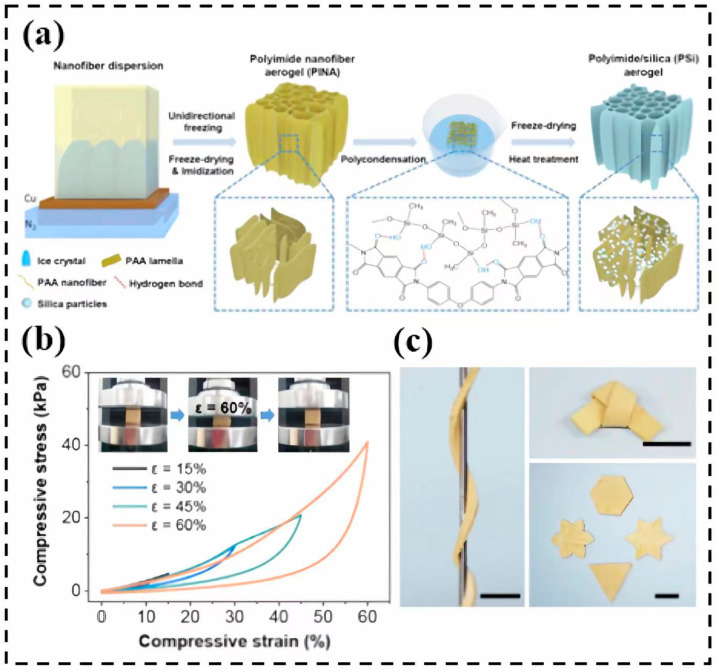
Preparation and composition of PSi aerogels: (**a**) schematic illustration of the preparation of PSi aerogels; (**b**) compressive stress–strain curves of PSi aerogel during loading–unloading cycles in the radial direction; (**c**) images of folded PSi-6 aerogel. Reprinted with permission from Ref. [143]: Copyright 2022, Elsevier.

Tian et al. [143] designed polyimide/silica composite aerogels using an integrated binary network via the in-situ synthesis. As shown in Figure 16a, the polyimide nanofiber aerogel (PINA) is used for the growth of the polymethylsilsesquioxane (PMSQ) network to synthesize the polyimide/silica (PSi) composite aerogel. The composite aerogels demonstrate excellent compressibility and flexibility, recovering from large compression (ε = 60%) and showing no collapsing. Meanwhile, the stress–strain curves (Figure 16b) demonstrate excellent compression recovery properties from 15%, 30%, 45% and 60% strain, respectively. In addition, the thermal conductivity of the polyimide/silica aerogel is as low as 0.0212 W/m·K and shows excellent resistance under 1200 °C.

**Figure 17:**
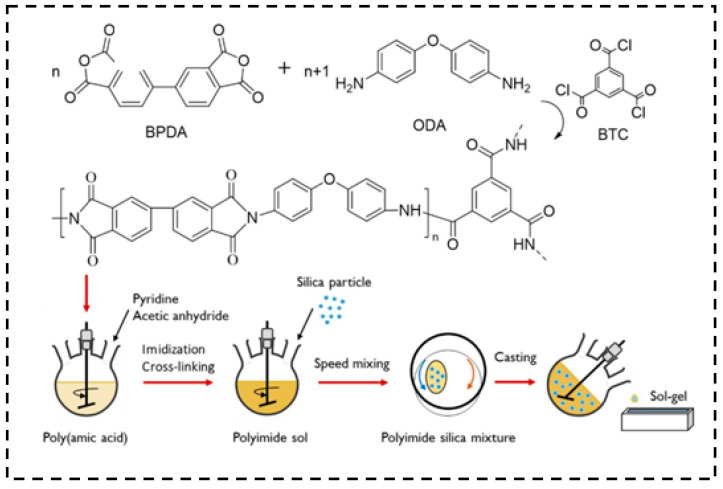
The preparation of PI-silica composites. Reprinted with permission from Ref. [144]: Copyright 2022, Elsevier.

Kantor et al. [144] synthesized heterogeneous polyimide–silica aerogels with low shrinkage by adding silica aerogel particles into a polyimide sol. The polyimide–silica aerogels exhibited heterogeneous structures and have properties of a high surface area over 609 m^2^/g and a low thermal conductivity of 0.0175 W/m·K. Compared with general polyimide materials, the composite aerogels demonstrated potential commercial value. As shown in Figure 17, the BTC solution and silica aerogel powders occurred gelation in the polypropylene container, and the samples were obtained through aging and supercritical drying.

Fei et al. [145] prepared the polyimide-crosslinked silica aerogels using different weight percentages of polyimide via the condensation reaction. The thermal conductivity of the aerogel is as low as 0.0306~0.0347 W/m·K, and the relatively high compressive strength can achieve a 1.03~3.82 MPa. Besides using polyimide to crosslink with silica aerogels to enhance their mechanical properties, using polyimide as a reinforcing phase is an efficient technical approach to improve the mechanical properties of silica aerogels. Fei et al. [146] used glass fiber and polyimide (PI) to reinforce the silica aerogel.

Zhang et al. [147] applied the “co-gel” strategy to fabricate the novel silica/polyimide (SiO_2_/PI) nanocomposites. The SiO_2_/PI nanocomposite aerogels exhibited excellent mechanical properties due to the hierarchically porous structure, such as a compressive modulus (1.96 MPa) and specific modulus (52.7 m^2^/s^2^). Moreover, the materials exhibited excellent flame resistance and low thermal conductivities between 25 °C and 300 °C.

### 3.5. Polystyrene

Polystyrene (PS) is a non-polar material that can improve the hydrophobic properties of silica aerogels. The silica precursors modified via amine, vinyl, and AIBN were also used to crosslink with PS to prepare the PS-reinforced silica aerogels.

Ilhan et al. [148] designed a new three-dimensional core–shell structure in which the PS was applied as the shell via the method of the free-radical polymerization process. Compared to the composite material prepared via polyurea and epoxy, the PS-crosslinked silica aerogels demonstrate a better hydrophobicity. Moreover, the silica aerogels showed excellent mechanical properties and maintained their integrity, while the thermal conductivity was as low as 0.041 W/m·K.

Maleki et al. [149] applied the growth of grafted polymers from the surface of silica gel to prepare the mechanically reinforced polymer–silica aerogels. The method of surface-initiated reversible addition–fragmentation chain transfer polymerization can significantly improve the compression strength of silica aerogels. Matias et al. [87] used polybutylacrylate (PBA) and polystyrene (PS) to prepare crosslinked flexible, monolithic, and superhydrophobic silica aerogels. Compared with the non-reinforced aerogel, the PBA-reinforced aerogel, MTMS-derived aerogel, and PS-reinforced aerogel demonstrated excellent Young’s modulus values and compression strength, which can reach 91 kPa and 68 kPa, respectively. DeFriend et al. [150] used polystyrene beads to prepare mesoporous silica aerogel to investigate the influences of the surface area and pore volume on mechanical compression. These results demonstrate that the templating agents had a great effect on the compressive strength of the aerogels and that the concentrations were a great factor.

## 4. Conclusions

In this review, the synthesis chemistry and three main stages were introduced. Then, the process of sol–gel chemistry and the role of aging were also described in detail. The advantages and disadvantages of the three drying methods listed were also carefully analyzed and compared.

The five common polymers used to enhance the mechanical properties of silica aerogels were summarized. In the process of modification, the crosslinking agent has an important function. More importantly, the improvement in the mechanical properties is significantly influenced by process parameters such as time, temperature, and ratio. The linear density and shrinkage of the material increases significantly when the process parameters are not well controlled. This inevitably makes the aerogel lose the original advantages and causes a reduction in the mechanical properties. The silica aerogels’ crosslinked polymers demonstrated good mechanical properties. In particular, polystyrene demonstrated a better performance of hydrophobicity due to its characteristics.

## 5. Outlook and Prospects

Compared with physical strengthening, the bonding tightness of silica aerogel particles strengthened via polymer crosslinking has the advantages of firmness and reliability. However, some problems still need to be further studied in the field of polymer reinforcement. Researchers should not only focus on the influence of polymers on the thermo-mechanical properties of aerogels during the research process, but should also carefully consider the effects of crosslinked polymers on other aspects of silica aerogels, such as the increase in density due to the addition of polymers, the poor flame retardancy of some polymers, and the difficult aging resistance of hybrid aerogels. The technology of eliminating the negative effects of polymers should be carefully studied. The functional role of silica in various fields should also be given more attention. Polymer crosslinked silica aerogels fully demonstrate the lightweight porous properties of aerogels and the related properties of polymers. Thus, this work will lay a solid and reliable foundation for the future development of multifunctional hybrid aerogels.

## Figures and Tables

**Table 2:** Overview of reported mechanical properties of polymer-reinforced aerogels.

Precursor Formulation	Polymer Matrix	Enhanced Properties	Ref
GPTMS/VTMS	Epoxide	➢ Elastic deformation: 3~5%	[128]
/	Epoxide	➢ Elastic modulus: 35%, tensile strength: 62%, toughness: 126%	[129]
/	Epoxide	➢ (Hydrophobic aerogel) contact angles: 107°, fracture toughness: improved by up to ∼70%, impact strength: improved by up to ∼120%	[130]
/	Epoxide	➢ Elastic modulus: 3770 ± 71 MPa, stress at yield point: 43.2 ± 1.8 MPa, strain at yield point: 1.24 ± 0.03 MPa, ultimate tensile strength: 51.0 ± 2.1 MPa, strain at break point: 3.3 ± 0.3%, toughness: 1.29 ± 0.08 J/m^3^	[131]
TEOS/APTES	Epoxide	➢ Strain: 80% (18 N)	[132]
TEOS/APTES	Epoxide	➢ Tensile strength: 45.05± 4.56 MPa, modulus of elasticity: 4363.88± 209.57 MPa, break strain: 1.19 ± 0.17%	[133]
TEOS	Epoxide	➢ Density: 0.419 g/cm^3^, porosity: 89%, compressive strength: 0.438 MPa, thermal conductivity: 0.0273 W/m·K	[134]
/	Epoxide	➢ (Warp direction) strength: 464.3 MPa, modulus: 1.76 GPa, (weft direction) strength: 410.2 MPa, modulus: 1.68 GPa	[135]
TMOS	Polyurea	➢ Shrinkage: 14.6 ± 0.7%, bulk density: 0.594 ± 0.026 g/cm^3^, skeletal density: 1.290 ± 0.003 g/cm^3^, porosity: 54%	[136]
TEOS/APTES	Polyurea	➢ Bulk density: 0.046 g/cm^3^, flexural modulus: 0.14 MPa	[137]
TEOS/APTES	Polyurea	➢ Linear shrinkage: 15.73%, bulk density: 0.392 g/m^3^, average elastic modulus: 14.57 MPa	[138]
TEOS /MTEOS	Polyurethane	➢ Density: 0.190 ± 0.006 g/m^3^, yield strength: 2.15 ± 0.04 MPa, Young’s modulus: 50 ± 0.09 MPa	[139]
/	Polyurethane	➢ Heat resistance index: 193.6%, char yield: 31.6%, bulk density: 0.580 g/mL	[140]
TEOS/APTES	Polyurethane	➢ BET surface area: 242.9 m^2^/g, BJH desorption average pore diameter: 10.8 nm	[141]
TEOS	Polyurethane	➢ Density: 117.68 kg/m^3^, porosity: 92.3%, linear shrinkage: −8.38%, thermal conductivity: 0.014 ± 0.00033 W/m·K	[142]
MTMS	Polyimide	➢ Compressive strain: 50%, thermal conductivity: 0.0212 W/m·K	[143]
/	Polyimide	➢ Surface area: 609 m^2^/g, thermal conductivity: 0.017.5 W/m·K	[144]
TEOS/APTES	Polyimide	➢ Compressive strength: 3.82 MPa, Young’s modulus: 44.16 MPa	[145]
TEOS/APTES	Polyimide	➢ Density: 0.145 g/cm^3^, strain: 9%, strength: 0.29 MPa, Young’s modulus: 3.22 MPa	[146]
TEOS	Polyimide	➢ Compressive modulus:1.96 MPa, thermal conductivity: 0.0311~0.0585 W/m·K	[147]
TMOS/APTES	Polystyrene	➢ Density: 0.41~0.77 g/cm^3^, surface area: 213~393 m^2^/g, thermal conductivity: 0.041 W/m·K, contact angles: 120°	[148]
TMOS	Polystyrene	➢ Density: 0.13~0.17 g/cm^3^, surface area: 350~780 m^2^/g, thermal conductivity: 0.03~0.04 W/m·K	[149]
MTMS/VTMS/TMOS	Polystyrene	➢ Bulk density: 163.1 ± 11.7 kg/cm^3^, porosity: 88%, surface area: 227 m^2^/g, thermal conductivity: 0.072 ± 0.001 W/m·K, Young’s modulus: 91 kPa, compression strength: 68 kPa	[87]
TMOS	Polystyrene	➢ Modulus: 3 MPa	[150]

## Data Availability

Not applicable.
